# Decreased Endometrial Thickness Is Associated With Higher Risk of Neonatal Complications in Women With Polycystic Ovary Syndrome

**DOI:** 10.3389/fendo.2021.766601

**Published:** 2021-11-29

**Authors:** Jialyu Huang, Jiaying Lin, Leizhen Xia, Lifeng Tian, Dingfei Xu, Peipei Liu, Jing Zhu, Qiongfang Wu

**Affiliations:** ^1^ Center for Reproductive Medicine, Jiangxi Maternal and Child Health Hospital, Nanchang University School of Medicine, Nanchang, China; ^2^ Department of Assisted Reproduction, Shanghai Ninth People’s Hospital, Shanghai Jiao Tong University School of Medicine, Shanghai, China; ^3^ Center for Reproductive Medicine, The First Affiliated Hospital of Wenzhou Medical University, Wenzhou, China

**Keywords:** endometrial thickness, obstetric outcome, neonatal outcome, polycystic ovary syndrome, embryo transfer

## Abstract

**Purpose:**

To evaluate the association of endometrial thickness (EMT) with obstetric and neonatal outcomes in women with polycystic ovary syndrome (PCOS).

**Methods:**

A total of 1755 subfertile PCOS women with singleton livebirths after frozen-thawed embryo transfer were included between January 2009 and September 2019. Main obstetric outcomes were hypertensive disorders in pregnancy and abnormal placentation. Main neonatal outcomes were preterm birth (PTB), low birthweight (LBW) and small-for-gestational age (SGA). Crude and adjusted odds ratios (ORs) with 95% confidence intervals (CIs) were estimated by univariate and multivariate logistic regression analyses.

**Results:**

Each millimeter decrease in EMT was related to a 9% (adjusted OR 1.09, 95% CI 1.00–1.19; *P* = 0.053), 14% (adjusted OR 1.14, 95% CI 1.02–1.28; *P* = 0.002) and 22% (adjusted OR 1.22, 95% CI 1.07–1.38; *P* = 0.003) higher risk of PTB, LBW and SGA, respectively. Compared to women with EMT >13 mm, women with EMT ≤8 mm also had significantly higher risk of PTB (adjusted OR 3.79, 95% CI 1.53–9.39; *P* = 0.004), LBW (adjusted OR 4.33, 95% CI 1.39–13.50; *P* = 0.012) and SGA (adjusted OR 6.38, 95% CI 1.78–22.83; *P* = 0.004). These associations remained consistent in further subgroup analysis by endometrial preparation regimen and in sensitivity analyses among nulligravida women or women without adverse obstetric outcomes. No significant differences were found in the incidence of several pregnancy complications across EMT categories.

**Conclusion:**

Decreased EMT was independently associated with increased risk of PTB, LBW and SGA in women with PCOS.

## Introduction

Polycystic ovary syndrome (PCOS) is a common endocrine disorder that affects 4–21% of reproductive-aged women worldwide (1). This heterogeneous syndrome is the major cause of female anovulatory infertility and is associated with increased risk of complications during pregnancy and perinatal period (2–4). Higher incidences of gestational diabetes mellitus (GDM), pregnancy-induced hypertension (PIH), pre-eclampsia (PE) and cesarean section (CS) have been documented in pregnant women with PCOS, while neonates born to them are more likely to suffer from preterm birth (PTB), small-for-gestational age (SGA) and admission to intensive care units (2–4).

The pathophysiology of obstetric and neonatal complications in PCOS is not entirely understood, but has been suggested to be related to primary disease characteristics such as hyperandrogenism, insulin resistance and obesity (4, 5). Recent studies have also indicated an abnormal endometrial phenotype and function in women with PCOS (6–8), which may lead to impaired decidual endovascular trophoblast invasion and defective placentation process associated with adverse pregnancy outcomes (9–11). Compared with normal endometrium, the PCOS endometrium has been observed to overexpress androgen receptors and fail to downregulate estrogen receptor-α during the secretory phase (12, 13). Conversely, the expression of endometrial receptivity markers, including αvβ3 integrin, homeobox A10 and insulin-like growth factor-binding protein 1, is decreased in the window of implantation (14–16). Nonetheless, whether these markers predict clinical outcomes in PCOS women is still poorly investigated (8).

In addition to endometrial biopsy for molecular evaluation, measurement of endometrial thickness (EMT) *via* transvaginal ultrasound (TVU) is non-invasive and routinely performed during infertility treatment to assess uterine receptivity. Piles of evidence have demonstrated that decreased peak EMT is associated with a lower pregnancy chance in both fresh and frozen-thawed embryo transfer (FET) cycles (17–19). More recently, women with thin endometrial linings were also found to have increased odds of obstetric disorders and reduced neonatal birthweight (20–25). These findings suggest that EMT might be an important indicator of placentation and fetal development, while its predictive value in PCOS deserves further clarification.

The aim of the present study was to evaluate the relationship of EMT with obstetric and neonatal outcomes in women with PCOS.

## Materials and Methods

### Study Design and Participants

This single-center retrospective cohort study was conducted at the Department of Assisted Reproduction, Shanghai Ninth People’s Hospital affiliated with Shanghai Jiao Tong University School of Medicine. The study protocol was approved by the Institutional Review Board of the hospital, and written informed consents for data collection and research use were obtained from all couples prior to treatment initiation.

Subfertile women with PCOS who underwent FET cycles and had singleton livebirths after ≥24 weeks of gestation were enrolled from January 2009 to September 2019. The diagnosis of PCOS was in accordance with the revised Rotterdam criteria, which require the presence of at least 2 of the following 3 features: clinical and/or biochemical hyperandrogenism, oligo- or anovulation, and polycystic ovarian morphology on ultrasound, with exclusion of other etiologies (26). Analyses were restricted to FET cycles to eliminate the confounding effect of supraphysiological estradiol (E_2_) level in fresh embryo transfer (27–29), while multiple births were excluded for their well-established association with pregnancy complications (30). Other exclusion criteria were: maternal smoking; congenital uterine malformations; acquired uterine diseases including adenomyosis, submucosal fibroids, intrauterine adhesions and endometrial polyps; and cycles with missing data or lost to follow-up. In cases of more than one delivery from the same woman during the study period, only the first livebirth was included for analysis.

### Ovarian Stimulation and Laboratory Protocols

Controlled ovarian stimulation was performed using gonadotropin-releasing hormone (GnRH) agonist, GnRH antagonist or progestin (P) for pituitary suppression (31). The initial dose of human menopausal gonadotropin (hMG) was dependent on patient characteristics, with subsequent adjustment made according to ovarian response. Ovulation was triggered as soon as one leading follicle size reached 20 mm or three follicles were ≥18 mm. Oocyte retrieval took place 34 to 36 hours later, and collected oocytes were inseminated by *in vitro* fertilization (IVF), intracytoplasmic sperm injection (ICSI) or both based on semen quality. Embryos were cultured up to day 3 or day 5/6, and graded for morphology according to the Cummins’s criteria or Gardner and Schoolcraft’s scale, respectively (32, 33). Cleavage-stage embryos of grade I/II and blastocyst-stage embryos of grade 3BC or higher were classified as high quality and chosen for vitrification.

Sequential embryo culture with Early Cleavage Medium and Multi-Blast Medium (Irvine Scientific, USA) was applied in our center before 2013, while Continuous Single Culture (Irvine Scientific, USA) was used thereafter. Except for this switch, other clinical procedures and laboratory protocols were unaltered, and have been described in detail in our previous publication (31).

### Endometrial Preparation and Thickness Measurement

All PCOS women underwent hormone therapy cycle or ovulation induction cycle for endometrial preparation (34). Since no consensus has been reached on the best FET cycle regimen (35), the decision was made through a combination of physician discretion and patient preference.

In hormone therapy cycle, oral administration of E_2_ (Fematon-red tablets, Abbott Biologicals, USA) was started from day 3 of the spontaneous or induced menstrual cycle, at a daily dose of 6 mg for 2 weeks. When necessary, the dose of E_2_ could be increased to 8 mg *per* day with an extended use for another week. Exogeneous P was given both vaginally (400 mg/d; Utrogestan, Besins Manufacturing, Belgium) and orally (dydrogesterone 40 mg/d; Fematon-yellow tablets), after which cleavage-stage or blastocyst-stage embryos were transferred 3 or 5 days later, respectively.

In ovulation induction cycle, letrozole 5 mg *per* day was commenced on the 3rd day of menstruation for 5 days. From cycle day 10 onwards, follicular monitoring was initiated using TVU and serum hormone measurement. When the leading follicle size was below 14 mm on the 10th day, a low-dose hMG (75 IU/d; Anhui Fengyuan Pharmaceutical Co., China) could be injected for further stimulation. Ovulation was induced with 5000 IU human chorionic gonadotropin (hCG) (Lizhu Pharmaceutical Trading Co., China), as soon as the diameter of dominant follicle exceeded 17 mm with serum E_2_ >150 pg/mL and P <1 ng/mL. The administration of hCG was performed at 9:00 p.m. if luteinizing hormone (LH) level was less than 20 mIU/mL, or immediately in cases of LH surge ≥20 mIU/mL. Vaginal micronized P (400 mg/d; Utrogestan) was supplemented following the presumed day of ovulation, with embryos transferred 3 or 5 days later according to the developmental stage. In both cycle regimens, luteal phase support was continued until 10 weeks of gestation when a pregnancy was achieved.

To guarantee the accuracy and reliability as possible, endometrial lining thickness was measured by highly trained physicians with at least 7 years’ TVU experience using a 6-MHz vaginal transducer (Voluson; GE Healthcare, Austria). In our center, a patient was assigned to the same physician for the whole process of IVF treatment in order to keep a consistent monitoring and reduce female discomfort with TVU by different operators. We identified EMT as the widest distance between the reflective interfaces of the endometrium and the myometrium of opposite sides in midsagittal plane of the uterus. In hormone therapy cycle, peak EMT was recorded on the last TVU conducted before P provision, while EMT taken on the hCG trigger day was used in ovulation induction cycle.

### Outcome Measures

The primary neonatal outcomes were PTB, low birthweight (LBW) and SGA. Other neonatal outcomes included very PTB, postterm birth, very LBW, macrosomia, large-for-gestational age (LGA), major birth defects and neonatal hospitalization for more than 3 days. We defined PTB, very PTB and postterm birth as gestational age <37, <32 and ≥42 weeks, respectively. LBW, very LBW and macrosomia were identified as birthweight <2500, <1500 and ≥4000 grams, respectively. Based on the singleton growth standard of Chinese population (36), we further calculated birthweight Z-score after adjusting for gestational week and infant sex, and defined SGA and LGA as birthweight <10th and >90th percentiles, respectively. Birth defects were categorized according to the International Classification of Diseases (ICD)-10 codes Q00-Q99, and were considered as major if they generally cause functional impairment or require surgical correction (37).

We also assessed obstetric complications in women with singleton livebirths, which included vanishing twin syndrome (VTS), hypertensive disorders in pregnancy (HDP), GDM (ICD-10 code O24.4), placenta previa (ICD-10 code O44), placenta accreta (ICD-10 code O43.2), placental abruption (ICD-10 code O45), preterm premature rupture of membranes (PPROM) (ICD-10 code O42), postpartum hemorrhage (PPH) (ICD-10 code O72) and CS (ICD-10 code O82). VTS was defined as clinical pregnancy that underwent spontaneous reduction from ≥2 gestational sacs at 7 weeks’ gestation to singleton delivery eventually (38). HDP in this study comprised PIH (ICD-10 code O13), PE (ICD-10 code O14) and eclampsia (ICD-10 code O15) as a whole (39).

### Statistical Analysis

Women were stratified into three groups on the basis of peak EMT during FET cycle: ≤8, >8 to ≤13 and >13 mm. This division corresponds to the 10th (8.0 mm) and 90th (13.2 mm) percentiles of EMT distribution across the study population, and is also consistent with threshold values used in previous studies (23, 24, 40, 41). For continuous variables, data were presented as mean ± standard deviation and analyzed by one-way analysis of variance (for normal distribution) or Kruskal-Wallis test (for skewed distribution). Categorical variables were described as number and percentage, with the use of Chi-square test or Fisher’s exact test to compare differences among groups.

We conducted multivariate logistic regression analysis to assess the independent association of EMT with PTB, LBW and SGA risks. The following variables were considered as potential confounders: maternal age (in years), body mass index (<18.5, 18.5–24.9, 25.0–29.9 or ≥30 kg/m^2^), gravidity (0, 1, 2 or ≥3), parity (0 or ≥1), history of PTB (yes or no), history of CS (yes or no), infertility duration (in years), infertility diagnosis (PCOS only, PCOS + male factor, PCOS + tubal factor, PCOS + male/tubal factors or PCOS + other factors), FET cycle rank (1, 2 or ≥3), fertilization method (IVF, ICSI or IVF + ICSI), endometrial preparation regimen (hormone therapy cycle or ovulation induction cycle), duration of embryo cryopreservation (in years), embryo transfer number (1 or 2), embryo developmental stage (cleavage or blastocyst) and year of treatment (before 2013 or after 2013). EMT was separately introduced in the regression model as a continuous variable (model 1) or a categorical variable (model 2). The results were computed as crude and adjusted odds ratios (ORs) with 95% confidence intervals (CIs).

Data analyses were performed using SPSS 20.0 (SPSS Inc., USA) and MedCalc 15.0 (MedCalc Software bvba, Belgium). A two-sided *P*-value <0.05 was considered as statistically significant.

## Results

### Patient Characteristics

In total, we analyzed 1755 PCOS women who satisfied the study criteria, including 184 (10.5%) women with EMT ≤8 mm, 1379 (78.6%) with EMT >8–13 mm, and 192 (10.9%) with EMT >13 mm. Peak EMT in the study cohort ranged from 4.6 to 21.9 mm, and average values for the three groups were 7.38 ± 0.66, 10.25 ± 1.28 and 14.56 ± 1.52 mm, respectively (*P <*0.001) (**Figure S1**).

Patient baseline demographics and cycle characteristics are presented in **Table 1**. The distribution of gravidity and endometrial preparation regimen varied significantly among the three EMT categories (both *P <*0.001). Conversely, no significant differences were found among the groups in terms of maternal age, body mass index, parity, history of PTB or CS, infertility duration or diagnosis, rank of FET cycle, fertilization method, duration of embryo cryopreservation, number of embryos transferred, embryo developmental stage and year of treatment.

**Table 1:** Baseline demographics and cycle characteristics grouped by endometrial thickness.

	≤8 mm (*n* = 184)	>8 to ≤13 mm (*n* = 1379)	>13 mm (*n* = 192)	*P*-value
Maternal age (years)	29.9 ± 3.3	30.0 ± 3.5	30.1 ± 3.5	0.992
Maternal BMI (kg/m^2^)	23.44 ± 3.70	23.45 ± 3.85	24.02 ± 4.26	0.343
Gravidity, *n* (%)				<0.001
0	92 (50.0)	922 (66.9)	140 (72.9)	
1	58 (31.5)	319 (23.1)	39 (20.3)	
2	22 (12.0)	93 (6.7)	7 (3.6)	
≥3	12 (6.5)	45 (3.3)	6 (3.1)	
Parity, *n* (%)				0.141
0	180 (97.8)	1333 (96.7)	181 (94.3)	
≥1	4 (2.2)	46 (3.3)	11 (5.7)	
Prior preterm birth, *n* (%)	1 (0.5)	5 (0.4)	1 (0.5)	0.462
Previous cesarean section, *n* (%)	3 (1.6)	20 (1.5)	3 (1.6)	0.978
Duration of infertility (years)	3.1 ± 2.6	3.5 ± 2.6	3.4 ± 2.4	0.094
Infertility diagnosis, *n* (%)				0.086
PCOS only	69 (37.5)	408 (29.6)	48 (25.0)	
PCOS + male factor	21 (11.4)	166 (12.0)	30 (15.6)	
PCOS + tubal factor	82 (44.6)	686 (49.7)	99 (51.6)	
PCOS + male/tubal factors	7 (3.8)	91 (6.6)	14 (7.3)	
PCOS + other factors	5 (2.7)	28 (2.0)	1 (0.5)	
Rank of cycle, *n* (%)				0.388
1	115 (62.5)	851 (61.7)	118 (61.5)	
2	37 (20.1)	342 (24.8)	51 (26.6)	
≥3	32 (17.4)	186 (13.5)	23 (12.0)	
Fertilization method, *n* (%)				0.251
IVF	117 (63.6)	820 (59.5)	119 (62.0)	
ICSI	30 (16.3)	288 (20.9)	45 (23.4)	
IVF + ICSI	37 (20.1)	271 (19.7)	28 (14.6)	
Endometrial preparation regimen, *n* (%)				<0.001
Hormone therapy cycle	99 (53.8)	577 (41.8)	46 (24.0)	
Ovulation induction cycle	85 (46.2)	802 (58.2)	146 (76.0)	
Duration of embryo cryopreservation (years)	0.46 ± 0.46	0.46 ± 0.52	0.50 ± 0.68	0.573
Embryo transfer number, *n* (%)				0.226
Single	37 (20.1)	226 (16.4)	26 (13.5)	
Double	147 (79.9)	1153 (83.6)	166 (86.5)	
Embryo stage at transfer, *n* (%)				0.055
Cleavage	148 (80.4)	1181 (85.6)	171 (89.1)	
Blastocyst	36 (19.6)	198 (14.4)	21 (10.9)	
Year of treatment, *n* (%)				0.994
Before 2013	14 (7.6)	108 (7.8)	15 (7.8)	
After 2013	170 (92.4)	1271 (92.2)	177 (92.2)	

Data are presented as mean ± standard deviation or number (percentage).

BMI, body mass index; ICSI, intracytoplasmic sperm injection; IVF, in vitro fertilization; PCOS, polycystic ovary syndrome.

### Obstetric Outcomes

**Table 2** shows the obstetric outcomes according to EMT stratification. There was a tendency towards a higher PPROM risk in women with EMT ≤8 mm compared to those with EMT >8–13 mm and EMT >13 mm (3.8% *vs.* 2.8% and 1.0%, respectively), whereas the difference failed to reach statistical significance (*P* = 0.184). Similarly, a non-significant increase was also observed in the rate of abnormal placentation composing placenta previa, placenta accreta and placental abruption (2.2% *vs.* 1.5% and 0.5%, respectively; *P* = 0.347). The proportion of HDP (7.6% *vs.* 5.2% and 5.7%; *P* = 0.409) was comparable among the three groups, along with other pregnancy complications including VTS, GDM, PPH and CS.

**Table 2:** Obstetric outcomes grouped by endometrial thickness.

	≤8 mm (*n* = 184)	>8 to ≤13 mm (*n* = 1379)	>13 mm (*n* = 192)	*P*-value
Vanishing twin syndrome	14 (7.6)	94 (6.8)	14 (7.3)	0.907
Hypertensive disorders in pregnancy	14 (7.6)	72 (5.2)	11 (5.7)	0.409
Gestational diabetes mellitus	27 (14.7)	175 (12.7)	24 (12.5)	0.742
Abnormal placentation	4 (2.2)	20 (1.5)	1 (0.5)	0.347
Placenta previa	3 (1.6)	17 (1.2)	1 (0.5)	0.544
Placenta accreta	1 (0.5)	3 (0.2)	0 (0)	0.407
Placental abruption	0 (0)	1 (0.1)	0 (0)	1.000
Preterm premature rupture of membranes	7 (3.8)	38 (2.8)	2 (1.0)	0.184
Postpartum hemorrhage	0 (0)	7 (0.5)	1 (0.5)	1.000
Cesarean section	135 (73.4)	1002 (72.7)	133 (69.3)	0.585

Data are presented as mean ± standard deviation or number (percentage).

### Neonatal Outcomes

The outcomes of live-born singleton infants are summarized in **Table 3**. Although gestational age at delivery seemed unaltered by EMT categories (38.54 ± 2.12 *vs.* 38.72 ± 1.85 and 38.97 ± 1.39 weeks, respectively; *P* = 0.369), the incidence of PTB was significantly higher in women with EMT ≤8 mm than in women with EMT >8–13 mm and EMT >13 mm (13.6% *vs.* 9.3% and 3.6%, respectively; *P* = 0.003). Birthweight (3260.1 ± 632.3 *vs.* 3314.6 ± 545.4 and 3443.1 ± 439.4 g, respectively; *P* = 0.004) and its Z-score (0.33 ± 1.18 *vs.* 0.39 ± 1.07 and 0.61 ± 0.98, respectively; *P* = 0.006) were also significantly lower in the EMT ≤8 mm group, consistent with the increased frequency of LBW (9.2% *vs.* 5.6% and 2.1%, respectively; *P* = 0.010) and SGA (9.2% *vs.* 4.3% and 1.6%, respectively; *P* = 0.001). We did not find statistically significant differences regarding the rates of male gender, very PTB, postterm birth, very LBW, macrosomia, LGA, major birth defects and neonatal hospitalization >3 days.

**Table 3:** Neonatal outcomes grouped by endometrial thickness.

	≤8 mm (*n* = 184)	>8 to ≤13 mm (*n* = 1379)	>13 mm (*n* = 192)	*P*-value
Male gender, *n* (%)	91 (49.5)	706 (51.2)	92 (47.9)	0.656
Gestational age (weeks)	38.54 ± 2.12	38.72 ± 1.85	38.97 ± 1.39	0.369
Preterm birth, *n* (%)	25 (13.6)	128 (9.3)	7 (3.6)	0.003
Very preterm birth, *n* (%)	3 (1.6)	22 (1.6)	2 (1.0)	0.822
Postterm birth, *n* (%)	0 (0)	1 (0.1)	1 (0.5)	0.383
Birthweight (g)	3260.1 ± 632.3	3314.6 ± 545.4	3443.1 ± 439.4	0.004
Low birthweight, *n* (%)	17 (9.2)	77 (5.6)	4 (2.1)	0.010
Very low birthweight, *n* (%)	4 (2.2)	16 (1.2)	0 (0)	0.055
Macrosomia, *n* (%)	15 (8.2)	109 (7.9)	12 (6.2)	0.708
Birthweight Z-score	0.33 ± 1.18	0.39 ± 1.07	0.61 ± 0.98	0.006
Small-for-gestational age, *n* (%)	17 (9.2)	59 (4.3)	3 (1.6)	0.001
Large-for-gestational age, *n* (%)	37 (20.1)	242 (17.5)	40 (20.8)	0.419
Neonatal hospitalization >3 days, *n* (%)	13 (7.1)	71 (5.1)	4 (2.1)	0.077
Major birth defects, *n* (%)	2 (1.1)	11 (0.8)	1 (0.5)	0.824

Data are presented as mean ± standard deviation or number (percentage).

### Multivariate Analysis

Multiple logistic regression models were further employed to investigate the association between EMT and adverse neonatal outcomes in women with PCOS. As demonstrated in **Table 4**, *per* 1 mm decrease of EMT led to 9% (adjusted OR 1.09, 95% CI 1.00–1.19; *P* = 0.053), 14% (adjusted OR 1.14, 95% CI 1.02–1.28; *P* = 0.002) and 22% (adjusted OR 1.22, 95% CI 1.07–1.38; *P* = 0.003) greater likelihood of developing PTB, LBW and SGA, respectively. Compared to women with EMT >13 mm as the reference, women with EMT ≤8 mm also had significantly higher risk of incident PTB (adjusted OR 3.79, 95% CI 1.53–9.39; *P* = 0.004), LBW (adjusted OR 4.33, 95% CI 1.39–13.50; *P* = 0.012) and SGA (adjusted OR 6.38, 95% CI 1.78–22.83; *P* = 0.004).

**Table 4:** Crude and adjusted analyses of PTB, LBW and SGA based on endometrial thickness.

	Crude OR (95% CI)	*P*-value	Adjusted OR (95% CI)	*P*-value
Preterm birth (*per* 1 mm decrease)	1.11 (1.03–1.21)	0.011	1.09 (1.00–1.19)	0.053
≤8 mm	4.16 (1.75–9.86)	0.001	3.79 (1.53–9.39)	0.004
>8–13 mm	2.70 (1.24–5.88)	0.012	2.71 (1.22–6.04)	0.015
>13 mm	Reference		Reference	
Low birthweight (*per* 1 mm decrease)	1.15 (1.04–1.28)	0.010	1.14 (1.02–1.28)	0.022
≤8 mm	4.78 (1.58–14.50)	0.006	4.33 (1.39–13.50)	0.012
>8–13 mm	2.78 (1.01–7.68)	0.049	2.70 (0.97–7.55)	0.058
>13 mm	Reference		Reference	
Small-for-gestational age (*per* 1 mm decrease)	1.20 (1.07–1.36)	0.003	1.22 (1.07–1.38)	0.003
≤8 mm	6.41 (1.85–22.27)	0.003	6.38 (1.78–22.83)	0.004
>8–13 mm	2.82 (0.87–9.07)	0.083	2.87 (0.88–9.36)	0.081
>13 mm	Reference		Reference	

CI, confidence interval; LBW, low birthweight; OR, odds ratio; PTB, preterm birth; SGA, small-for-gestational age.

For sensitivity analysis, we restricted the outcomes of singletons to nulligravida women (**Table S1**) and women without obstetric complications (**Table S2**), and found similarly significant association of EMT with PTB, LBW and SGA. Subgroup analysis was also performed according to endometrial preparation regimen given its uneven distribution among the different EMT categories (**Table S3**). The adjusted ORs for SGA remained statistically significant after stratification, although the risks of PTB and LBW were attenuated and became marginally significant or non-significant.

## Discussion

Based on a large sample size of 1755 live-born singletons following FET cycles, this retrospective cohort study showed that decreased EMT was independently associated with higher risk of PTB, LBW and SGA in women with PCOS. There was no significant difference in the incidence of several obstetric complications across EMT categories, while an upward trend was found among PCOS women with thin endometrial linings.

The relationship of EMT with obstetric and perinatal outcomes has been previously investigated in women with unspecified infertility diagnosis. The first study by Chung et al. (20) reported that each millimeter decrease in EMT was related to a 12% relative increase in the risk of PTB, LBW and intrauterine fatal demise occurring beyond the first trimester. This finding was subsequently confirmed by Moffat et al. (21) showing that EMT was positively correlated with neonatal birthweight and duration of gestation. In 2018, Oron et al. (22) demonstrated an elevated incidence of composite obstetric complications in women with peak EMT <7.5 mm (adjusted OR 1.53, 95% CI 1.03–2.42), while no significant difference was detected in separate analysis for each outcome including PE, placental abruption, manual removal of adherent placenta and SGA. By retrospectively analyzing 939 and 6181 singleton pregnancies in fresh and FET cycle, two other studies also observed that reduced EMT was associated with lower birthweight Z-score, although the adjusted ORs of PTB, LBW and SGA failed to reach statistical significance (23, 24). Contrarily, in the recent study by Guo et al. (25), women with thin endometrial linings were found to have a nearly 2.5-fold higher risk of delivering SGA infants (adjusted OR 2.39, 95% CI 1.16–4.95). Despite inconsistency in individual results, these studies all highlight the potential effect of EMT on placentation and further fetal development.

In the present study, particular attention was paid to PCOS as it represents *per se* a risk factor for adverse maternal and perinatal outcomes (2–4). The median value of EMT in PCOS women was 10.2 mm, which appears to be similar to that in general subfertile women (10.3 mm) after analysis of 17244 FET pregnancy cycles in our center (42). This comparability is contradictory to previous studies that reported increased thickness of PCOS endometrium due to continuous unopposed estrogen action (43–45), but could be possibly because all women underwent oocyte retrieval and had withdrawal bleeding prior to FET. Compared to women with unspecified infertility indications (20–25), we found a consistently significant but seemingly stronger association between EMT and neonatal complications in women diagnosed with PCOS.

Similar to previous reports (20–25), our study has determined the cut-off points in an arbitrary approach based on the EMT percentage distribution of included patients as well as the general definition of thin endometrium (46). For the purpose of practical guidance, restricted cubic splines with four knots were additionally used to map out the risk of neonatal complications throughout different values of EMT (47). As demonstrated in **Figure S2**, the probability of PTB, LBW and SGA all increased with a decreased EMT among PCOS women with EMT <8 mm, but was constantly lower in women with EMT >8 mm. This finding, together with our regression analysis results, suggests that an EMT of 8 mm may be used a clinically meaningful cut-off point, while more studies are needed for further verification.

The underlying mechanisms for this relationship are still unclear. One possible explanation may lie in the role of oxygen tension. In the presence of thin endometrial linings, the embryos could be implanted closer to the spiral arteries of the endometrial basal layer, where the higher vascularity and oxygen levels may result in accumulation of reactive oxygen species and thus pose a detrimental impact on embryos (48, 49). In addition, the spiral arteries are physiologically remodeled into uteroplacental vessels early during gestation and this process involves both the decidual and junctional zone myometrial segments (50). Therefore, a thin endometrium might cause incomplete transformation and eventually lead to defective deep placentation associated with a spectrum of pregnancy complications (50, 51). However, further investigations are warranted to examine these speculations with stronger and more direct evidence.

Intriguingly, a tendency towards a higher PPROM risk was observed among PCOS women with thin endometria. To date, only two studies have reported the association between EMT and PROM (52, 53). Martel et al. (52) found that the incidence of PROM was comparable between women with an EMT of <7 mm and those with an EMT of ≥7 mm (0% *vs.* 0.2%). The study included a total of 492 patients who had singleton livebirths after single euploid embryo transfer in a hormone therapy FET cycle, but only 7 patients were in the EMT<7 mm group. In another study by He et al. (53), the prevalence of PROM was also higher in women with EMT <8 mm than in women with EMT ≥8 mm [6.7% (10/150) *vs.* 5.1% (50/989)], whereas the difference was not statistically significant (*P* = 0.410). Pre-pregnancy intrauterine infection has been suggested to be an important cause of thin endometria (46), while persistent or potential infection during pregnancy is likely to be a crucial reason for PPROM (54). Therefore, we speculate that the influence of infectious factors may contribute to this possible relationship, but further studies should be conducted to explore the detailed mechanism for clinical implications.

A major strength of our study is the robustness of results after performing multivariate regression and sensitivity/subgroup analyses according to gravidity, obstetrical complication and endometrial preparation regimen. Another notable advantage is that we evaluated EMT separately as a continuous and a categorical variable. In most previous studies, a thin endometrium was conventionally defined as peak EMT below 7, 7.5 or 8 mm to facilitate the application of findings in clinical practice (22–25). However, the stratification of continuous variables may limit the assessment of the real effect of a predictor as it assumes that values in different categories have different influences even if close to each other, and that values on the extremes, but within the same category, have the same influence (23). Therefore, two regression models were applied in the present study and their consistent results further confirmed the significant association of EMT with PTB, LBW and SGA risks among PCOS women.

Despite our caution, there are still several limitations that merit consideration. Firstly, this study is retrospective in nature and not all confounding factors have been included for adjustment, such as social-economic status, phenotypic variants of PCOS and metabolic patterns before pregnancy (4, 5). Specially, insulin resistance has been suggested to be intrinsic of PCOS and play an important role in its pathogenesis and development of pregnancy complications including GDM, PIH and PE (9, 55–57). However, related data were unavailable for the lack of records in our electronic database. Only FET cycles were included for analysis, which should caution the generalization of study conclusion to PCOS women who had natural conceptions or underwent other assisted reproductive treatment including fresh embryo transfer, intrauterine insemination and ovulation induction. Secondly, maternal and neonatal follow-up surveys were mainly conducted by trained nurses through telephone calls without direct access to medical records (58). Therefore, the diagnosis of obstetric diseases could not be uniformed according to the latest criteria and their prevalence may be underestimated or overestimated. Separate analyses on the indication of CS and classification of HDP were also unavailable for the lack of information in our standardized questionnaire. Thirdly, while the sample size is comparable to some of the largest PCOS birth cohorts to date (5), only ~10% of women were identified with EMT ≤8 mm and the relatively small subset of cases resulted in wide confidence intervals. In addition, our study may still have insufficient statistical power to detect potentially clinically important differences in adverse obstetric outcomes with low incidence. Finally, although endometrial assessment was performed by the same team of experienced physicians in our center, ultrasound images were not saved for further validation of EMT and the intra- and inter-observer inconsistency could be an insurmountable source of bias (59). During the long study period, the resolution of ultrasonography could be improved due to technique advancement (Voluson 730/E6/E8) and may thus affect the accuracy of EMT. In this regard, further prospective cohort studies with cooperative ultrasound evaluation by an expert group using the same machine types should be conducted to confirm our conclusion.

In conclusion, our study demonstrated that decreased EMT was an independent risk factor for PTB, LBW and SGA in PCOS. This novel finding suggests that EMT may be applied as a simple indicator of neonatal complications among women with PCOS. For the purpose of better infant health, more clinical strategies should be proposed to optimize endometrial development, while increased surveillance should be provided to pregnant PCOS women with thin endometrium.

## Data Availability Statement

The original contributions presented in the study are included in the article/**Supplementary Material**. Further inquiries can be directed to the corresponding authors.

## Ethics Statement

The studies involving human participants were reviewed and approved by the Ethics Committee of Shanghai Ninth People’s Hospital affiliated with Shanghai Jiao Tong University School of Medicine. The patients/participants provided their written informed consent to participate in this study.

## Author Contributions

JH, JZ, and QW contributed to the conception and design of the study. JH and JL were responsible for data collection and manuscript drafting. LX and LT conducted the statistical analyses. DX and PL were involved in data interpretation and discussion. JZ and QW. supervised the project administration. All authors read and approved the final manuscript.

## Funding

This study was funded by the National Natural Science Foundation of China (81960288).

## Conflict of Interest

The authors declare that the research was conducted in the absence of any commercial or financial relationships that could be construed as a potential conflict of interest.

## Publisher’s Note

All claims expressed in this article are solely those of the authors and do not necessarily represent those of their affiliated organizations, or those of the publisher, the editors and the reviewers. Any product that may be evaluated in this article, or claim that may be made by its manufacturer, is not guaranteed or endorsed by the publisher.

## Supplementary Material

Supplementary Figure 1
**(A)** Distribution of endometrial thickness in the study cohort. **(B)** Stratification of endometrial thickness by the 10th and 90th percentiles. Horizontal lines represent mean value (blue) and standard deviation (green).

Supplementary Figure 2 Predicted probability of **(A)** preterm birth (PTB), **(B)** low birthweight (LBW), and **(C)** small-for-gestational age (SGA) by endometrial thickness (mm) after adjustment for covariates. The shaded region represents the 95% confidence intervals.
