# Supplementary figures and images for: Decreased Endometrial Thickness Is Associated With Higher Risk of Neonatal Complications in Women With Polycystic Ovary Syndrome
Source: Front Endocrinol (Lausanne). 2021 Nov 29;12:766601. doi: 10.3389/fendo.2021.766601 (PMC8667169; doi:10.3389/fendo.2021.766601)

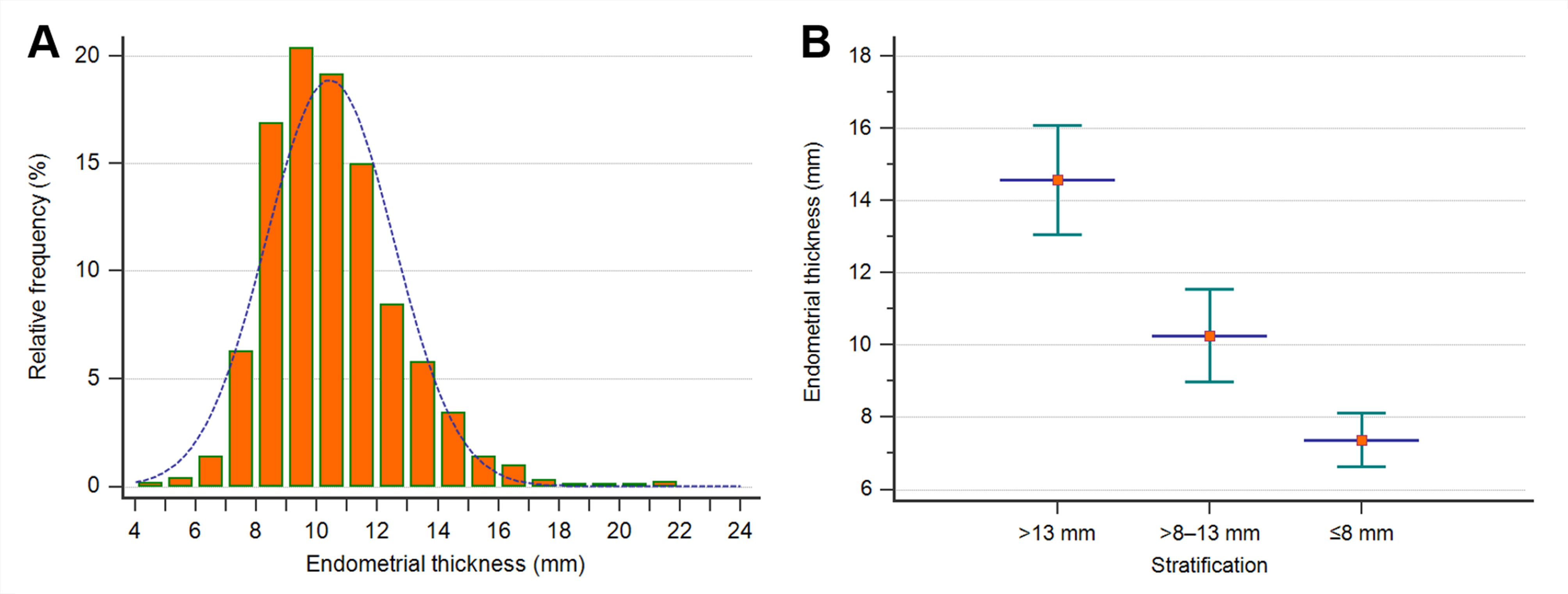

Supplement: Supplementary Figure 1 — (A) Distribution of endometrial thickness in the study cohort. (B) Stratification of endometrial thickness by the 10th and 90th percentiles. Horizontal lines represent mean value (blue) and standard deviation (green). [file Image_1.tif]

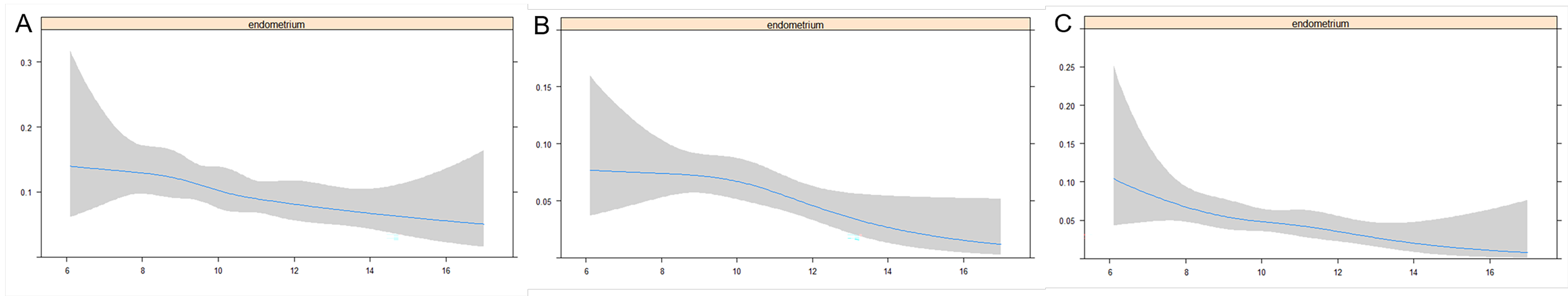

Supplement: Supplementary Figure 2 — Predicted probability of (A) preterm birth (PTB), (B) low birthweight (LBW), and (C) small-for-gestational age (SGA) by endometrial thickness (mm) after adjustment for covariates. The shaded region represents the 95% confidence intervals. [file Image_2.tif]
