# Supplementary material for: Decreased Endometrial Thickness Is Associated With Higher Risk of Neonatal Complications in Women With Polycystic Ovary Syndrome
Source: Front Endocrinol (Lausanne). 2021 Nov 29;12:766601. doi: 10.3389/fendo.2021.766601 (PMC8667169; doi:10.3389/fendo.2021.766601)
Supplement: Supplementary file 3 [file Table_1.docx]

**Table S1.** Association of endometrial thickness with PTB, LBW and SGA risks in nulligravida women.

|  | Model 1^a^ |  | Model 2^b^ | | |
| --- | --- | --- | --- | --- | --- |
|  | All |  | ≤8 mm | >8 to ≤13 mm | >13 mm |
| Preterm birth |  |  |  |  |  |
| *n/N* (%) | 108/1154 (9.4) |  | 16/92 (17.4) | 87/922 (9.4) | 5/140 (3.6) |
| Crude OR (95% CI) | 1.15 (1.04–1.28) |  | 5.68 (2.00–16.13) | 2.81 (1.12–7.06) | Reference |
| *P*-value | 0.009 |  | 0.001 | 0.027 | – |
| Adjusted OR (95% CI) | 1.11 (1.00–1.24) |  | 4.74 (1.63–13.80) | 2.64 (1.04–6.68) | Reference |
| *P*-value | 0.055 |  | 0.004 | 0.041 | – |
| Low birthweight |  |  |  |  |  |
| *n/N* (%) | 70/1154 (6.1) |  | 9/92 (9.8) | 58/922 (6.3) | 3/140 (2.1) |
| Crude OR (95% CI) | 1.20 (1.05–1.37) |  | 4.95 (1.30–18.81) | 3.07 (0.95–9.92) | Reference |
| *P*-value | 0.006 |  | 0.019 | 0.062 | – |
| Adjusted OR (95% CI) | 1.18 (1.03–1.35) |  | 4.27 (1.09–16.74) | 2.86 (0.87–9.35) | Reference |
| *P*-value | 0.018 |  | 0.037 | 0.083 | – |
| Small-for-gestational age |  |  |  |  |  |
| *n/N* (%) | 51/1154 (4.4) |  | 5/92 (5.4) | 44/922 (4.8) | 2/140 (1.4) |
| Crude OR (95% CI) | 1.15 (0.99–1.33) |  | 3.97 (0.75–20.89) | 3.46 (0.83–14.43) | Reference |
| *P*-value | 0.070 |  | 0.104 | 0.089 | – |
| Adjusted OR (95% CI) | 1.18 (1.01–1.38) |  | 4.71 (0.86–25.81) | 3.68 (0.87–15.63) | Reference |
| *P*-value | 0.037 |  | 0.074 | 0.078 | – |

^a^ Endometrial thickness as a continuous variable (*per* 1 mm decrease).

^b^ Endometrial thickness as a categorical variable.

Abbreviations: CI, confidence interval; LBW, low birthweight; OR, odds ratio; PTB, preterm birth; SGA, small-for-gestational age.

**Table S2.** Association of endometrial thickness with PTB, LBW and SGA risks in women without obstetric complications.

|  | Model 1^a^ |  | Model 2^b^ | | |
| --- | --- | --- | --- | --- | --- |
|  | All |  | ≤8 mm | >8 to ≤13 mm | >13 mm |
| Preterm birth |  |  |  |  |  |
| *n/N* (%) | 122/1334 (9.1) |  | 21/139 (15.1) | 96/1051 (9.1) | 5/144 (3.5) |
| Crude OR (95% CI) | 1.11 (1.01–1.22) |  | 4.95 (1.81–13.53) | 2.80 (1.12–6.99) | Reference |
| *P*-value | 0.033 |  | 0.002 | 0.028 | – |
| Adjusted OR (95% CI) | 1.09 (0.99–1.21) |  | 4.88 (1.70–13.98) | 2.93 (1.14–7.55) | Reference |
| *P*-value | 0.085 |  | 0.003 | 0.026 | – |
| Low birthweight |  |  |  |  |  |
| *n/N* (%) | 74/1334 (5.5) |  | 14/139 (10.1) | 57/1051 (5.4) | 3/144 (2.1) |
| Crude OR (95% CI) | 1.14 (1.01–1.29) |  | 5.26 (1.48–18.74) | 2.70 (0.83–8.72) | Reference |
| *P*-value | 0.036 |  | 0.010 | 0.098 | – |
| Adjusted OR (95% CI) | 1.12 (0.99–1.27) |  | 4.29 (1.19–16.21) | 2.57 (0.78–8.46) | Reference |
| *P*-value | 0.082 |  | 0.026 | 0.120 | – |
| Small-for-gestational age |  |  |  |  |  |
| *n/N* (%) | 57/1334 (4.3) |  | 13/139 (9.4) | 42/1051 (4.0) | 2/144 (1.4) |
| Crude OR (95% CI) | 1.22 (1.05–1.41) |  | 7.33 (1.62–33.09) | 2.96 (0.71–12.34) | Reference |
| *P*-value | 0.008 |  | 0.010 | 0.137 | – |
| Adjusted OR (95% CI) | 1.24 (1.06–1.45) |  | 7.47 (1.57–35.51) | 2.91 (0.68–12.49) | Reference |
| *P*-value | 0.006 |  | 0.011 | 0.150 | – |

^a^ Endometrial thickness as a continuous variable (*per* 1 mm decrease).

^b^ Endometrial thickness as a categorical variable.

Obstetric complications included vanishing twin syndrome, hypertensive disorders in pregnancy, gestational diabetes mellitus, placenta previa, placenta accreta and placental abruption. Abbreviations: CI, confidence interval; LBW, low birthweight; OR, odds ratio; PTB, preterm birth; SGA, small-for-gestational age.

**Table S3.** Subgroup analysis of PTB, LBW and SGA according to endometrial preparation regimen.

|  | Model 1^a^ |  | Model 2^b^ | | |
| --- | --- | --- | --- | --- | --- |
|  | All |  | ≤8 mm | >8 to ≤13 mm | >13 mm |
| ***Hormone therapy cycle*** |  |  |  |  |  |
| Preterm birth |  |  |  |  |  |
| *n/N* (%) | 81/722 (11.2) |  | 18/99 (18.2) | 61/577 (10.6) | 2/46 (4.3) |
| Crude OR (95% CI) | 1.20 (1.04–1.37) |  | 4.89 (1.08–22.05) | 2.60 (0.62–11.00) | Reference |
| *P*-value | 0.010 |  | 0.039 | 0.194 | – |
| Adjusted OR (95% CI) | 1.22 (1.05–1.41) |  | 4.35 (0.93–20.41) | 2.32 (0.53–10.06) | Reference |
| *P*-value | 0.008 |  | 0.063 | 0.263 | – |
| Low birthweight |  |  |  |  |  |
| *n/N* (%) | 46/722 (6.4) |  | 12/99 (12.1) | 32/577 (5.5) | 2/46 (4.3) |
| Crude OR (95% CI) | 1.12 (0.95–1.33) |  | 3.03 (0.65–14.16) | 1.29 (0.30–5.57) | Reference |
| *P*-value | 0.181 |  | 0.158 | 0.731 | – |
| Adjusted OR (95% CI) | 1.15 (0.96–1.37) |  | 3.15 (0.64–15.42) | 1.25 (0.28–5.58) | Reference |
| *P*-value | 0.131 |  | 0.157 | 0.768 | – |
| Small-for-gestational age |  |  |  |  |  |
| *n/N* (%) | 29/722 (4.0) |  | 9/99 (9.1) | 20/577 (3.5) | 0/46 (0) |
| Crude OR (95% CI) | 1.29 (1.03–1.61) |  | N/A | N/A | Reference |
| *P*-value | 0.028 |  | – | – | – |
| Adjusted OR (95% CI) | 1.28 (1.01–1.61) |  | N/A | N/A | Reference |
| *P*-value | 0.042 |  | – | – | – |
| ***Ovulation induction cycle*** |  |  |  |  |  |
| Preterm birth |  |  |  |  |  |
| *n/N* (%) | 79/1033 (7.6) |  | 7/85 (8.2) | 67/802 (8.4) | 5/146 (3.4) |
| Crude OR (95% CI) | 1.03 (0.93–1.15) |  | 2.53 (0.78–8.24) | 2.57 (1.02–6.49) | Reference |
| *P*-value | 0.546 |  | 0.123 | 0.046 | – |
| Adjusted OR (95% CI) | 1.01 (0.91–1.13) |  | 2.21 (0.64–7.68) | 2.69 (1.03–6.98) | Reference |
| *P*-value | 0.891 |  | 0.210 | 0.043 | – |
| Low birthweight |  |  |  |  |  |
| *n/N* (%) | 52/1033 (5.0) |  | 5/85 (5.9) | 45/802 (5.6) | 2/146 (1.4) |
| Crude OR (95% CI) | 1.16 (1.01–1.34) |  | 4.50 (0.85–23.73) | 4.28 (1.03–17.84) | Reference |
| *P*-value | 0.042 |  | 0.076 | 0.046 | – |
| Adjusted OR (95% CI) | 1.12 (0.97–1.30) |  | 3.25 (0.59–18.01) | 4.25 (1.00–18.02) | Reference |
| *P*-value | 0.121 |  | 0.178 | 0.050 | – |
| Small-for-gestational age |  |  |  |  |  |
| *n/N* (%) | 50/1033 (4.8) |  | 8/85 (9.4) | 39/802 (4.9) | 3/146 (2.1) |
| Crude OR (95% CI) | 1.20 (1.03–1.39) |  | 4.95 (1.28–19.21) | 2.44 (0.73–7.99) | Reference |
| *P*-value | 0.019 |  | 0.021 | 0.142 | – |
| Adjusted OR (95% CI) | 1.20 (1.03–1.40) |  | 4.47 (1.08–18.56) | 2.42 (0.71–8.20) | Reference |
| *P*-value | 0.022 |  | 0.039 | 0.156 | – |

^a^ Endometrial thickness as a continuous variable (*per* 1 mm decrease).

^b^ Endometrial thickness as a categorical variable.

Abbreviations: CI, confidence interval; LBW, low birthweight; OR, odds ratio; PTB, preterm birth; SGA, small-for-gestational age.
